# Medical School Attrition-Beyond the Statistics A Ten Year Retrospective Study

**DOI:** 10.1186/1472-6920-13-13

**Published:** 2013-01-31

**Authors:** Bridget M Maher, Helen Hynes, Catherine Sweeney, Ali S Khashan, Margaret O’Rourke, Kieran Doran, Anne Harris, Siun O’ Flynn

**Affiliations:** 1 School of Medicine, College of Medicine and Health, Brookfield Health Sciences Complex, University College Cork, College Road, Cork, Ireland

**Keywords:** Medical school attrition, Dropout, Exit interviews, Student welfare services, Academic difficulty, Absenteeism

## Abstract

**Background:**

Medical school attrition is important - securing a place in medical school is difficult and a high attrition rate can affect the academic reputation of a medical school and staff morale. More important, however, are the personal consequences of dropout for the student. The aims of our study were to examine factors associated with attrition over a ten-year period (2001–2011) and to study the personal effects of dropout on individual students.

**Methods:**

The study included quantitative analysis of completed cohorts and qualitative analysis of ten-year data. Data were collected from individual student files, examination and admission records, exit interviews and staff interviews. Statistical analysis was carried out on five successive completed cohorts. Qualitative data from student files was transcribed and independently analysed by three authors. Data was coded and categorized and key themes were identified.

**Results:**

Overall attrition rate was **5.7%** (45/779) in 6 completed cohorts when students who transferred to other medical courses were excluded. Students from Kuwait and United Arab Emirates had the highest dropout rate (RR = 5.70, 95% Confidence Intervals 2.65 to 12.27;p < 0.0001) compared to Irish and EU students combined. North American students had a higher dropout rate than Irish and EU students; RR = 2.68 (1.09 to 6.58;p = 0.027) but this was not significant when transfers were excluded (RR = 1.32(0.38, 4.62);p = 0.75). Male students were more likely to dropout than females (RR 1.70, .93 to 3.11) but this was not significant (p = 0.079).

Absenteeism was documented in 30% of students, academic difficulty in 55.7%, social isolation in 20%, and psychological morbidity in 40% (higher than other studies). Qualitative analysis revealed recurrent themes of isolation, failure, and despair. Student Welfare services were only accessed by one-third of dropout students.

**Conclusions:**

While dropout is often multifactorial, certain red flag signals may alert us to risk of dropout including non-EU origin, academic struggling, absenteeism, social isolation, depression and leave of absence. Psychological morbidity amongst dropout students is high and Student Welfare services should be actively promoted. Absenteeism should prompt early intervention. Behind every dropout statistic lies a personal story. All medical schools have a duty of care to support students who leave the medical programme.

## Background

### *'Tread softly because you tread on my dreams’*

Throughout the world, places in medical school are highly-prized with a large number of applicants applying for a limited number of places. Although attrition from medical programmes is low compared to other university courses [1], every year students leave medicine, either by choice or by necessity. This has important consequences for the university, society, the profession and most importantly, the students themselves.

Having a low attrition rate is important, not least because of the difficulty in securing a place in medical school and the high cost of tax-funded medical training. For the medical profession, dropout results in a loss of useful contribution and impacts on medical workforce planning. A high attrition rate can affect the academic reputation of a medical school and staff morale and may have financial consequences with subsequent impact on research and teaching. Most important of all, however, is the effect of leaving medicine on the individual student - dropout has considerable financial, social and emotional consequences and can cause great distress, low morale and poor self-esteem.

Medical school attrition is therefore an important area in medical education. Research in this area can be used to inform medical school selection methods, teaching and curriculum, and student support services. The time-frame of this study covered a period of considerable change in our institution, including change in admission policies [2].

## Medical school attrition – current knowledge

### Attrition rate

A MEDLINE search identified 36 relevant studies (mainly US and UK, but also the Netherlands, Australia and South Africa). The studies varied in size, scope, design, variables, and methods of calculating dropout. A recent meta-analysis found an average attrition rate of 11.1% (range: 2.4–26.2%) [3] while a UK ten-year retrospective study found a dropout rate of 14% [4]. A retrospective cohort study at Nottingham University found an attrition rate of 6% [5].

### Factors associated with medical school attrition

Variables commonly studied in medical school attrition studies include age, gender, nationality, academic preparedness (prior academic performance, grade point averages) and medical school selection methods [3-16]. Besides ‘observable’ student variables such as gender, nationality and age, dropout may also be associated with ‘unobserved’ student characteristics such as commitment, resilience and motivation to study medicine and also with medical school (institution) factors (entry requirements, teaching, assessment procedures, curriculum design and delivery) [12]. There are a small number of studies (mainly Netherlands) on the impact of curriculum content and delivery on dropout [17] and a few studies (US, UK and Israel) [4,18,19] on the relationship between academic struggle at medical school and dropout. There are very few studies on physical/psychological morbidity [3] and little is known about the role of financial problems, relationship problems, homesickness, engagement with student welfare services, absenteeism and the effects of educational initiatives on dropout. Similarly, there is a lack of research on the effect of actual change in curriculum on dropout and on the personal effects of dropout on individual students.

We know, however, that there is no distinct ‘dropout profile’ and students leave medicine for a variety of reasons. Some leave because they have no choice (persistent academic failure) while others leave for non-academic reasons including change of mind about a medical career. Common reasons for dropout are persistent academic failure, wrong career choice, personal reasons, and physical and psychological illness [4]. However, academic preparedness has been the only consistent variable shown to be associated with dropout (US, UK, Australia, Netherlands) [3,6,8,9,11,12,14,20-25].

#### Academic preparedness

A lower mean A level grade was a significant predictor of medical student ‘struggling’ (the majority of whom dropped out) at Nottingham Medical School [7]. High grades in Biology, Chemistry and Physics (UK) [11], Mathematics [11], Biology [4,16] (UK, USA) and English (Australia) [14] were associated with lower dropout in some studies.

#### Socio-demographic factors

No specific socio-demographic variable has been found to be significantly associated with dropout [3].

##### Gender

While male gender has been linked with poorer performance in medical school, (UK, US) [7,9,26,27], data relating to dropout is conflicted - some studies found increased dropout in males (UK, Australia) [4,11,12] and others in females (US) [13-16,28]. Only three studies in O’Neill’s recent meta-analysis found an association between gender and dropout [3].

##### Age

Evidence of the effect of age is also inconsistent [4,7,11,12,14]. A Nottingham University dropout study did not find any relationship between age of entry and dropout [5].

##### Ethnicity

Not being white was found to be a significant predictor of struggling in UK students, especially in the pre-clinical years [5] and studies show that ethnic minority medical students have poorer academic performance (UK, US) [7,10,13,16,29,30]. However, ethnicity has not been shown to significantly influence dropout [3]. Many medical schools are increasing their intake of international students, and consequently research on the effects of ethnicity on dropout is important.

##### Social class

Previous studies have not found any consistent association between attrition and social class. Only one of the studies in O’Neill’s meta-analysis found an effect of social class on dropout [3,11,12].

#### Other student variables

##### Academic difficulty

Academic struggling [5], decelerated curriculum (US) [28], failing at least one basic science course in Year 1(US) and low Year 1 grade point averages [26] were strongly associated with dropout. Very few dropout studies have undertaken a detailed analysis of repeat examinations and repeat years.

##### Previous degree

Having a previous degree appears to be protective against dropout [11,12].

##### Social isolation

Social integration is an important determinant of student progression. A study in Western Australia found that students who had lower admission scores and who were shy and timid were less likely to complete the medical course than those with higher admission grades and who were more outgoing and uninhibited [8]. UK studies have shown that students living in on-campus accommodation had markedly lower dropout rates [7,8].

#### Institutional factors

##### Curriculum

Studies show higher dropout in traditional compared to problem-based learning curricula (UK, Netherlands) [4,17,31]. However, we could not identify any studies linking actual impact of change of curriculum on dropout (although change of curriculum has been linked with exam failure).

##### Selection processes

Overall, studies suggest that more targeted medical school selection processes rather than open admission are associated with lower dropout [22,32,33].

## Objectives

•To examine student factors associated with medical school attrition including previously unstudied variables.

•To study the personal and emotional effects of dropout on individual students.

•To examine the effects of institutional factors on dropout e.g. change in curriculum, new medical programmes.

## Methods

### Context

Our study was a retrospective descriptive study carried out at University College Cork looking at medical school attrition over a Ten-Year period (2001–2011). In line with international trends in medical education, a new Graduate-Entry programme was introduced during this period. This was also a decade that saw the introduction of problem-based learning medical curricula in many medical schools.

### Participants

The term ‘drop-out’ was used to describe students who failed to continue their medical studies including students who transferred to other medical schools (‘transfers’) during the period September 2001-August 2011. Dropout students were identified from archived medical school dropout files.

### Instruments/variables

#### Student files

Student files were the primary source of information (student demographic information, student-staff correspondence, inter-staff correspondence, examination grades, email records, staff observations, medical certificates, letters from sponsors and parents, clinical elective reports and transcripts of Student Welfare and student exit interviews (Table 1).

**Table 1:** Data recorded for each student

**Source and type of data**	
**University Admissions Office**	· Age at course entry
· Student number (unique student identifier generated at university entry).	· Age when left course
· Year started programme	· Total number of years on programme
· Name	· Type of programme (Direct-Entry, Graduate-Entry, Mature student).
· Address (country of origin)	· Previous degree
· Date of birth	**From University Examinations Office**
· Date of exit	· Last exam passed
· Year of programme student left	· Exam grades
· Gender	· Number of repeat years
· EU/non-EU	· Number times sat Autumn repeat examinations
	· Other degree awarded
	· Enrolment on another UCC programme (modules registered for examination)
	· Completed Medical Foundation Year (6 year programme)
**From Student File**	**Comment**
Marital status	Not usually documented.
Living at home	Not usually documented.
Evidence of English fluency problems	Likely to be under-reported.
Wrong course choice	Identified from exit interview, student comments and timing of withdrawal.
Parental influence in career choice	Unlikely to be documented. May be a ‘personal’ reason.
Student opinion that course too academically challenging	
Difficulty with programme as did not study science subjects previously	Because of course entry requirements, all Direct-Entry students have studied Higher Level Chemistry and at least one other science subject.
Documented evidence physical ill-health	Likely to be under-reported.
Documented evidence psychological illness	Depression, anxiety, personality disorders, eating disorders, stress-related illness.
	Likely to be under-reported.
Documented evidence financial problems	
Documented evidence substance abuse	Includes alcohol abuse. Likely to be under-reported.
Documented evidence family problems	
Evidence of home-sickness/not settling in	Likely to be under-reported.
Evidence of relationship problems	Likely to be under-reported.
Availed of student welfare service	Student visits to the student welfare service are recorded in student files. The student welfare service makes confidential referrals to the Student Health Centre (has a psychiatry clinic) and counseling services.
Proposed plans for the future	Identified at exit interview, file comments, or subsequent registered modules.
Formal exit interview	
Evidence of absenteeism	Likely to be non-reported or under-reported.
Evidence of academic difficulty	Examination failure. Low grades.
Left due to Two-Year Rule	
Staff comments/observations	Valuable observations were obtained from inter-staff correspondence/file entries.
Student comments	Identified from student correspondence/exit interview/file entries.
Reason for withdrawal	The specific over-riding reason for dropout was recorded if available.

#### Other sources of information

Data was also acquired from the Examinations Office, the Admissions Department, and the International Students’ Office (Table 1). Faculty staff were also interviewed (sensitive issues may not have always been entered in student files).

#### Exit interviews

All medical students who leave are invited to an exit interview (semi-structured interview) with the Head of Medical Education. These interviews (commenced 2005) seek to ascertain the predominant reason for leaving and future plans. In this study, exit interviews provided important information. Thematic analysis of student comments in exit interviews allowed insight into the effect of dropout on individual students.

#### Identification of variables

Based on the variables previously studied in international medical attrition studies, we compiled a list of variables at the initial study planning meetings. These variables included commonly studied variables such as gender, age, country of origin and previous degree and other variables of interest such as examination failure, evidence of physical illness, evidence of psychological illness, type of programme, and ‘wrong career choice.’

A random sample of twelve dropout files was coded by all three investigators responsible for data collection and entry (BM, CS, HH) and results were compared to ensure consistency. The file analysis required for the pilot coding brought recurrent issues, problems and patterns to our attention, prompting us to identify other variables with a view to giving more detailed information on students who left the medical programme. These variables included number of years in college, number of repeat examinations, number of repeat years, living at home, parental influence to study medicine, evidence of financial problems, evidence of family problems, relationship problems, future career plans, attendance at Student Welfare Service, evidence of social isolation, and evidence of English fluency problems (Table 1). Because our study had a large qualitative element, we considered that the inclusion of these variables, whilst perhaps not suitable for statistical analysis, would give valuable information on the reasons behind dropout and the effect of dropout on individual students.

##### Medical Programmes at University College Cork, Ireland

There are almost 1,000 medical students at UCC and these students come from diverse backgrounds and nationalities, in particular, North America and Malaysia. Current annual medical student intake is approximately 150 school-leaver (Direct-Entry) students, 60 Graduate-Entry students and 50 Malaysian students (a new partnership programme commenced with Malaysia and NUI Galway in September 2011 where students do pre-clinical training in UCC and return to Malaysia for clinical training).

***School-leaver (Direct-Entry) Programme:*** Students are selected based on a combination of the results of a State examination (Leaving Certificate Examination) usually taken at around the age of 18, combined with the results of an aptitude test (Health Professionals Admissions Test) [2].

***Graduate-Entry Programme***: Students must have obtained a second class honours degree (any discipline) and must have passed the GAMSAT test. This programme has approximately 55 places every year.

***Mature Entry Programme:*** These students (aged over 25) are selected at interview and join the 5-year school-leaver course (there are approximately three or four places every year).

**Course Structure**: The medical curriculum at UCC is an integrated systems-based curriculum. Basic sciences are taught in conjunction with clinical skills, professionalism, and research. From Year 3 onwards, students spend most of their time in full-time hospital and General Practice clinical electives. Progression on the course requires satisfactory completion of clinical rotations as well as passing written and practical exams. Successful progress also requires professional standards of behavior to be met at all times.

**Student Welfare Services** consist of:

•Personal mentoring with an assigned academic staff member (voluntary). After an initial meeting, subsequent meetings are at the request of the student.

•Access to senior faculty for specific issues/concerns.

•A student peer-support service.

•A formal Student Welfare Service (students self-refer or are referred by faculty staff). The Student Welfare Service can refer students to counselling services and to the Student Health Service (which has Outpatient Psychiatry clinics).

**Two-Year Rule:** Students who fail an end of year module have to repeat that exam in the Autumn, and if unsuccessful, have to sit the examination the following summer (repeat the year). Students must pass/progress within two academic years of first registration for each year of the programme and are allowed to repeat a year after failing a re-sit examination only twice during their studies. Students are not allowed two repeat years within the first three years of the programme. Students must complete their studies within seven years of registering for the first medical year. Students with termination notices have the right to appeal within a set time frame.

**Recent changes in Medical Programmes at UCC:** The curriculum changed from a traditional 6 year course to the current 5 year curriculum in the academic year 2005/2006. Medical school admission policy changed in September 2009. Current admission is now based on a combination of school leaving examination grades combined with the results of an aptitude test – the Health Professionals Admissions Test (HPAT) [2]. A four-year Graduate-Entry Programme was introduced in 2008. The first cohort graduated in June 2012.

### Procedure

Student files, examination records and other available information were analyzed with regard to the variables of interest. Coding of a variable as positive was conditional on the actual documentation of the presence of that variable in the student file i.e. presence of financial problems was coded as positive only if there was a file entry stating that financial problems existed. Similarly, absenteeism was only coded positive if there were file entries documenting student absences from clinics, lectures etc. Health problems and psychological problems often required an element of judgement but were only included if they were considered to have had a probable impact on the student’s progression (i.e. a student feeling stressed a few days before examinations would not be categorized as having a psychological/psychiatric disorder). In some of these cases, staff interviews helped to clarify the situation.

Variables were listed in the database as ‘evidence of physical ill-health, ‘evidence of social isolation’ etc. rather than ‘physical ill-health’ or ‘social isolation’, highlighting the importance of having documented proof and objective identification of the variable. The authors analyzing the files had no previous knowledge of these students, thence allowing assessment of the data without personal prejudice.

Examination failures, repeat years and leave of absence were all cross-checked with data from the Examinations Office. All data entries were reviewed by BM. Data omissions/ambiguities were clarified by reviewing student files and discussing with the research team. Decision was taken by consensus.

#### Data entry

Data related to students who left the medical programme September 2001- August 2011 was recorded in a secure customised database. Coded answers were supplemented by free text entries (Table 1).

#### Qualitative analysis

Student comments taken from student files and exit interviews, other interviews (Faculty), and student correspondence (letters and e-mails) were transcribed and independently analyzed by three authors with the aim of identifying common themes relating to the impact of dropout on individual students. Student comments were read and reread to immerse authors in the material. The data were then categorized and themes were identified from the categories. Finally, the authors compared and agreed themes. The authors who carried out the thematic analysis (BM, HH, CS) are experienced medical school lecturers.

### Analysis

#### Calculation of dropout rate

Dropout rate was calculated for the **completed cohorts 2002–2007** by expressing the number of dropout students as a percentage of the total number of students who enrolled during that time.

Dropout rate for the ten-year period 2001–2011 was calculated by dividing the total number of students who enrolled during that time period by the total number of students who dropped out during those 10 years (2001–2011). However, some of the dropout students included in this calculation may have started university before 2001 and there may be students who enrolled in the later years of the programme who may yet leave the programme.

#### Statistical analysis

The relationships between the students’ nationality, gender, type of programme (Graduate-Entry or school-leaver Direct-Entry) and risk of dropout were assessed by calculating the risk ratios and 95% confidence intervals (CI) in STATA 10.0. The relationship was considered to be statistically significant if the p-value was less than 0.05 and all tests were two-sided.

•Data on the entire 10-year period was used for descriptive and qualitative analysis.

•Data on five completed cohorts (2002–2007) was used for quantitative analysis (gender, nationality, type programme).

#### Ethical approval

Ethical approval was granted by the Cork Hospitals Research Ethics Committee (student consent was exempted due to the aggregation and anonymisation of data and the difficulty in obtaining retrospective consent).

## Results

### Attrition rate

Overall attrition rate (completed cohorts 2002–2007) was 6.8% (53/779) with rates ranging from 4.38% (2002/03) to 9.15% (2007/08) (Table 2). Eight of these dropout students left to continue their medical studies at another medical school (usually the student’s home country). Dropout rate was 5.7% (45/779) when these 8 transfer students were excluded (rates ranged from 4.38% (2002/03) to 7.74% in 2007/08). Current dropout rate for the 2008/09 cohort is 5.5% (excludes dropouts which may yet occur in the remaining six months of the students’ final year).

**Table 2:** Dropout rate completed Cohorts (2002–2007)

**Year started university**	**Total no. students**	**No. dropouts (%)**	**No. dropouts excluding transfers (%)**
Cohort 2002/03	114	5 (4.38%)	5 (4.38)
Cohort 2003/04	129	7 (5.42%)	5 (5.42)
Cohort 2004/05	134	9 (6.71%)	8 (5.97)
Cohort 2005/06	130	10 (7.69%)	9 (6.92)
Cohort 2006/07	130	9 (6.92%)	7 (5.38)
Cohort 2007/08	142	13 (9.15%)	11 (7.74)
Total	779	53 (6.8%)	45 (5.7)

Dropout rate in the ten-year period was 5.9% (81/1361) and 5.2% when transfer students were excluded.

### Gender

Analysis of 5 successive completed cohorts (September 2002–September 2006 inclusive) showed that males (22) were more likely to drop out than females (18); however, the relative risk was not significantly increased (RR = 1.70 [95% CI: 0.93, 3.11]; p = 0.079) (Table 3). When transfers were excluded, the relative risk decreased in males (RR = 1.58 [95% CI: 0.82, 3.05]: p = 0.165) (Table 4). The Ten-Year study gave similar results (Tables 5 and 6).

**Table 3:** Five -year completed cohort 2002–2006

**Variable**	**Dropout no.**	**Completed no.**	**Total no.**	**RR, 95% CIs**	**p-value**
Nationality	40	597	637	-	-
Irish + EU *	12	324	336	Reference	-
North American	7	66	73	2.68 (1.09, 6.58)	0.027
Malaysia + Singapore	7	142	149	1.31 (0.53, 3.27)	0.555
Kuwait + UAE^#^	11	43	54	5.70 (2.65, 12.27)	<0.0001
Other	3	22	25	3.36 (1.01, 11.13)	0.042
**Gender**	40	597	637		
Female	18	353	371	Reference	-
Male	22	244	266	1.70 (0.93, 3.11)	0.079
**Program**					
School-leaver (DE)	19	594	613	Reference	
Graduate-Entry	5	188	193	0.84 (0.32, 2.21)	0.72
Mature students	1	20	21		
Total	25	802	827		

*UAE* United Arab Emirates, *DE* Direct Entry.

**Table 4:** Five year completed cohort 2002–2006 (excluding transfer students)

**Variable**	**Dropout no.**	**Transfers**	**Completed no.**	**Total no.**	**RR, 95% CIs**	**p-value**
Nationality	34	6	597	637	-	-
Irish + EU *	11	1	324	336	Reference	-
North American	3	4	66	73	1.32 (0.38, 4.62)	0.756
Malaysia + Singapore	7	0	142	149		
Kuwait + UAE	11	0	43	54		
Other	2	1	22	25		
Gender	34	6	597	637		
Female	16	2	353	371	Reference	
Male	18	4	244	266	1.58 (0.82, 3.05)	0.165
Program	19	0	594	613		
School-leaver (DE)	3	2	188	193	Reference	
Graduate-Entry	0	1	20	21	0.51 (0.15, 1.69)	0.26
Total	22	3	802	827		

*UAE* United Arab Emirates, *DE* Direct Entry.

**Table 5:** Ten year dropout 2001–2011

**Variable**	**Drop out, n (%)**	**Completed n (%)**	**Total n**	**RR, 95% CIs**	**p-value**
Nationality	81 (5.9) Δ	1280 (94.1)	1361#	-	-
Irish + EU *	38 (4.5)	811 (95.5)	849	Reference [1]	-
North American	16 (9.5)	152 (90.5)	168	2.12 (1.21, 3.73)	0.008
Malaysia + Singapore	10 (4.3)	225 (95.7)	235	0.95(0.48, 1.88)	0.88
Kuwait + UAE	13 (19.1)	55 (80.9)	68	4.27 (2.39, 7.62)	<0.0001
Other	4 (9.8)	37 (90.2)	41	2.18 (0.82, 5.82)	0.12
Gender					
Female	37 (4.9)	722 (95.1)	759	Reference [1]	-
Male	44 (7.3)	558 (92.7)	602	1.50 (0.98, 2.29)	0.06

Δ Total number of students who dropped out during the period September 2001-August 2011.

# Total number of students who enrolled September 2001 – September 2011.

**Table 6:** Ten year dropout 2001–2011 (excluding students who transferred)

	**Drop out, n (%)**	**Completed, n (%)**	**Total, n**	**RR, 95% CIs**	**p-value**
Nationality	70 (5.2) Δ	1280 (94.8)	1350 #	-	-
Irish + EU	34	811	845	Reference [1]	-
North American	10	152	162	1.53 (0.77, 3.04)	0.22
Malaysia + Singapore	10	225	235	1.06 (0.53, 2.11)	0.87
Kuwait + UAE	13	55	68	4.75 (2.63, 8.57)	<0.0001
Other	3	37	40	1.86 (0.60, 5.81)	0.28
Gender					
Female	32 (4.2)	722 (95.8)	754	Reference [1]	-
Male	38 (6.4)	558 (93.6)	596	1.50 (0.95, 2.37)	0.08

Δ Number of students who dropped out excluding students who transferred during the period September 2001-August 2011.

# Total number of students who enrolled September 2001 – September 2011 excluding students who transferred.

### Nationality

Analysis of 5 successive completed cohorts found an increased rate of dropout in Kuwaiti and UAE students combined (RR = 5.70(2.65, 12.27); p < 0.0001). Irish and EU students demonstrated a low risk of dropout and were therefore used as the reference group (Table 3). There was increased risk of dropout in North American students (RR = 2.68 (1.09, 6.58); p = 0.027), but this was not significant when transfer students (Table 4) were excluded (RR = 1.32 (0.38, 4.62); p = 0.75). Similar results were found in the Ten-Year study (Tables 5 and 6).

### Type of programme

The Graduate-Entry Medicine programme commenced in 2008, thus we could only compare programmes for the past four years and numbers are small. Graduate-Entry students were less likely to drop out than school-leaver students (RR = 0.84 (0.32, 2.21) p = 0.72), even moreso when students who transferred were excluded (RR = 0.51, (0.15, 1.69), p = 0.26).

### Dropout and Age at university entry

The commonest age at entry to university of students who dropped out was 18, 20 and 21 (8 students in each category). The average entry age of all medical students during this period was 19 (SD 5.6 years).

### Year of medical programme and dropout

Over 60% dropout students were in First Year, with Year 3 having the next highest rate (16%). Final Year had the lowest rate (5%) (Table 7). Wrong career choice and academic difficulty were the commonest reasons for dropout in the early years of the programme (Table 5). Late dropout was associated with persistent academic failure, and psychological/physical ill-health. In many cases, multiple factors were associated with dropout and were often interlinked i.e. some students with academic difficulty may have left unexpectedly just before exam time.

**Table 7:** Dropout and year of study: cohorts 2002-2007

**Year started university**	**Year 1 Dropouts**	**Year 2 dropouts**	**Year 3 dropouts**	**Year 4 dropouts**	**Year 5 dropouts**	**Total no dropouts**
Cohort 2002/3	2	1		1	1	5
Cohort 2003/4	2	2	2	1		7
Cohort 2004/5	5	1	1	1	1	9
Cohort 2005/6	4	2	3		1	10
Cohort 2006/7	7	1	1			9
Cohort 2007/8	12	1				13
Total	**32 (60.3%)**	**8 (15%)**	**7 (16.6%)**	**3 (13%)**	**3 (5.6%)**	53

### Dropout according to year

#### Dropout rate completed cohorts (2002–2007)

The curriculum changed to an integrated systems-based curriculum in 2005/2006. Dropout rate that year (2005/6) increased by 1% (5.97% to 6.92%), but fell again the following year (2006/07) to 5.38% (Table 2). There was a rise in dropout (7.74%) the following year (2007/08, the year of highest dropout) with most of these being First Year students.

### *Factors associated with dropout - 10 year review*

Data relating to 70 students (excluding transfers) who dropped out of the medical programme over the ten-year period 2001–2011 was analysed.

### Academic difficulty

There was documented student file and examination results (repeating in autumn, repeating a year) evidence of academic difficulty in 55.7% of dropout students. Twenty dropout students (28%) were required to leave because of the Two-Year Rule reflecting consistent academic underperformance.

### Number of repeat years

Thirty-four dropout students (34/70; 48.5%) had to repeat at least 1 year. Examination records for the entire medical student population during that period show a yearly year repeat rate of 1.2% to 3.9%.

### Autumn repeat exams

Forty-six dropout students (65.7%) had to repeat exams in Autumn at least once. Recent examination records (2006–2011) show a yearly medical student Autumn repeat examination rate of 8.9%-13.4%.

### Documented absenteeism

There was student file evidence of absenteeism (from lectures, practicals, wards, or electives) as a cause for concern in 21 (30%) of dropout students. One student ‘disappeared without trace for weeks on end’. Absenteeism was associated with social isolation and academic failure in many cases.

### Leave of absence

Fourteen dropout students (20%) took one or more years leave of absence. Reasons were primarily health problems (including depression) and also family problems.

### Attendance at Student Welfare service

Twenty-four (34%) of students who dropped out had attended the Student Welfare service.

### Social isolation

Social isolation was documented in 20% of dropout student files and homesickness in 20%. Only one student who dropped out lived at home. Some students described themselves as ‘shy and withdrawn’ and not able to socialise. Many said they were lonely and had no friends. Social isolation was more prevalent in overseas students.

### English fluency problems

Four of the seven dropout students (10%) with documented English language problems in our study were from Kuwait.

### What courses did these students change to?

Seventeen (24%) of students who dropped out said they still wanted to study medicine and planned to apply elsewhere. Law was the most common alternate course (4). Other courses included Pharmacy, Mathematics, Engineering, Research, Nursing, other health courses, Science, Arts/Music and Teaching, Accountancy, Philosophy and the Navy. Reasons for withdrawal and subsequent career choices were not always evident in the student files.

### Medical school attrition – beyond the statistics

#### Qualitative analysis

Some students left without warning while others had been ‘under the radar’ for some time. The qualitative analysis highlighted some of the personal stories behind the statistics. Some were particularly poignant. The analysis identified a number of themes in keeping with the high prevalence of psychological ill-health (40%) and social isolation (20%) (Table 8): Loneliness, isolation, despair and fear for the future were common problems identified. Word frequency analysis identified words such as ‘lonely’, ‘isolated’ and ‘alone’. One student said that there had been a period when he hadn’t had a normal conversation for a month. Another student found it difficult to leave his apartment.

**Table 8:** Factors associated with attrition from the medical programme 2001–2011 (excluding students who transferred)

Factor *	**Number(%)**	**Comment**
Wrong career choice	26 (37)	Based on student file documentation of wrong course choice. Students aged 18/19 may lack the maturity to make informed career choices. Many students are unprepared for the volume of work or realise that they would be better suited to other careers.
Physical ill-health	10 (14)	There was a wide range of documented health problems ranging from uncontrolled diabetes, trauma (road traffic accidents), anorexia nervosa, tumours and mumps. Psychological problems often co-existed with physical morbidity.
Psychiatric/Psychological Morbidity †	28 (40)	One student took an overdose (recovered), one admitted to considering self-harm.
Depression	11 (16)	†Incidence of all these conditions including depression, anxiety, eating disorders and alcohol abuse may be much higher as these figures are based on documented diagnoses and do not reflect undiagnosed, unreported or undocumented cases.
Post-Traumatic Stress Disorder	2 (3)	
Psychosis	2 (3)	
Eating disorders †	2 (3)	
Substance abuse †	2 (3)	

Homesickness	14 (20)	Homesickness and ‘difficulty settling in’ also affected Irish students. In one case, ‘homesickness by proxy’ was a factor (the student left because of a spouse’s homesickness).
Social isolation	14 (20)	Some students had very little contact with other students. One student rarely left his apartment. Students mentioned being shy and withdrawn, not being able to socialise, being lonely, having no friends and having no-one to talk to.
Family/personal problems	13 (18)	
Financial problems	10 (14)	Some students with large loans were worried that they would have to repay substantial sponsorship amounts and some even feared imprisonment when they returned home. Other students took leave of absence specifically to earn money for loan repayment.
English fluency problems	7 (10)	This was based on documented file evidence and is likely to be higher. 4 of these students were from Kuwait, 2 from Malaysia and 1 from UAE.
Relationship issues	5 (7)	Usually related to a student leaving to live near their partner rather than relationship break-ups.
Parental pressure to study medicine	1	File evidence of only 1 case, however, likely to be under-reported and may have been classifued as ‘personal’.
Accommodation problems	5 (7)	Under-reporting likely. Problems cited related to noisy house-mates, frequent house parties, untidy apartments.

*Dropout is often multi-factorial. The incidence of all factors is likely to be under-reported.

‘I have nobody to relate to and to share my thoughts and problems with’.

‘*It has been difficult to make friends, more so because of my personality as I am a very quiet and shy person’.*

‘I think I am in deep trouble and my future is in jeopardy. I have no one to talk to’.

Feelings of failure, loss and regret were highlighted by several students and many felt that they had disappointed family and sponsors.

‘*I feel that I am losing the biggest chance I ever had.’*

‘Medicine is my dream and it's the only thing I want to do in my life’.

‘I hope you can understand how difficult a decision this has been for me as medicine has been a lifelong ambition’.

Lack of self-sufficiency and difficulty in coping were also identified. One student only ate take-away food and another student admitted not being able to use the University virtual learning platform.

‘The problems have been building up inside of me’.

The analysis also gave a sense of the sacrifices made by individual students to study medicine. Some had ended up in major financial debt. Some students with large loans were worried that they would have to repay substantial sponsorship amounts and feared imprisonment when they returned to their native country. Other students took leave of absence specifically to earn money for loan repayment.

Wrong course choice was supported by a variety of student disclosures.

‘I am not able to go back into the pressure of medical school’.

‘This isn’t for me after all, I realize that now’.

‘I hate this clinical stuff’.

‘I was most unhappy in the course and I think it best if I find something that suits me better’.

‘I didn’t realize that there would be so much work involved’.

‘Growing up, my passion was something else and I sacrificed that dream in the hopes of establishing myself as a doctor. However, I now realize that it was not the right decision’.

## Discussion

### Main findings

•Non-EU students (Kuwait and United Arab Emirates) were at increased risk of dropout (p = 0.0001). North Americans were also at increased risk (p = 0.008) but this was not significant when transfers were excluded (p = 0.75). Malaysian students were not at increased risk of dropout.

•Males were more likely to drop out than females but this difference was not significant.

•There was a lower rate of dropout in Graduate-Entry students but this was not statistically significant.

•Change of curriculum was associated with a transient and small increase in dropout in the first year of the new curriculum.

•Dropout can have serious emotional, financial and personal consequences for medical students.

•There was a high prevalence of pyschological/psychiatric problems in dropout students. However, most dropout students did not attend Student Welfare or other supports.

•Social isolation was common.

•There was a high rate of documented absenteeism in students who dropped out.

•Sixty per cent of dropouts occurred in First Year, 16% in Third Year and 5% in Final Year.

## Discussion and comparison with other studies

### Country of origin

While studies have shown that ethnic minority medical students are at increased risk of academic ‘struggling’ [5,7,13,16,29,30] and one study found increased risk of dropout [16], our study found an increased risk of dropout in students from Kuwait and UAE but not Malaysia (this country-specific effect has not been noted before). Academic admission criteria for Kuwaiti and Malaysian students are comparable and both cohorts are government sponsored. The higher number of Malaysian students, however, may protect against social isolation. Language fluency problems may also be a contributory factor.

The low dropout rate of Malaysian students is of particular interest in view of the large numbers of Malaysian students who travel abroad to study Medicine.

North Americans were also at increased risk of dropout (p = 0.008) but this was not significant when transfer students were excluded (p = 0.75).

### Gender

Similar to other studies [4,11,12], our study found that while males were at increased risk of dropout, this was not significant.

### Type of programme

Similar to other studies [17,31,34], the Graduate-Entry programme had a lower dropout rate, moreso when students who transfered were excluded. However, our numbers are small and it will be interesting to see future analyses in this area. Graduate-Entry programmes are more likely to utilise Problem-Based Learning (PBL) which is associated with lower dropout than traditional curricula [4,17,31].

### Institutional changes - change in curriculum

While studies have found higher dropout in students studying a traditional curriculum than a PBL curriculum [4,17,31], there has been little research on effects of curriculum change.

Our curriculum changed to an integrated systems-based curriculum in 2005/2006. Dropout rate that year (2005/6) increased by 1% (5.97 to 6.92), but fell again the following year (2006/07) to 5.38%. There was a rise in dropout (7.74%) the following year (2007/08, the year of highest dropout) with most of these being First Year students. The increase in dropout in 2005/06 may be due to a small, but transient effect of curriculum change or may have been circumstantial, suggesting that faculty adjust quickly to delivering a new curriculum and that change in curriculum does not adversely affect the student experience. If increased dropout numbers were simply organizational or ‘teething problems’ of a new curriculum, one would also have expected higher dropout numbers in 06/07 which was not the case. One would expect that a new curriculum might impact during the first few years when students who have to repeat exams or perhaps repeat a year, find themselves ‘falling between two curriculae’. The majority of dropouts in the 2007/08 cohort (third year of new curriculum) were First Years (may be related to unidentified institutional factors or may be a chance occurrence).

#### Change in selection methods

Selection methods changed in September 2009. While it is too early to study the effects of these changes (cohorts admitted under the new admission criteria have not yet graduated), dropout in First Year students since September 2009 has been low. However, there is insufficent evidence as yet to link the recent decline in dropout with change in medical school selection methods.

### Other factors associated with attrition

We identified a number of variables associated with dropout including examination failure, psychological problems (40%), absenteeism (30%), homesickness (20%), English fluency problems (10%) and social isolation (20%).

#### Academic difficulty

Our findings agree with previous research linking academic difficulty and dropout [26,34,35]. Unsurprisingly, there was evidence of academic difficulty in over half the dropout students. The percentage of dropout students who repeated at least one year was 48.5% (average medical student yearly rate of 3.2%). Similarly, 65.7% of dropout students had to sit an autumn repeat examination on one or more occasions (compared with an average medical student exam repeat rate of 16%).

Interventions to improve academic performance and study skills may help decrease dropout in this easily identifiable group. However, examination grades can also be an effect of underlying problems -academic strugglers may not be well integrated academically or socially and this may affect their commitment to the programme as hypothesised by Tinto’s interactional model of student attrition [36].

#### Psychological problems

Our findings of psychological/psychiatric morbidity in 40% of dropout students is higher than other studies [4,6,8,23] and a cause for concern. It is likely that the prevalence of psychological problems/depression is even higher than documented, as students are often reluctant to seek help. It is sometimes unclear whether such psychological symptoms are the cause of, or the result of, academic failure. Our study found little file information on alcohol abuse, despite our awareness of this being a significant problem in our student population. Medicine as a career requires good physical and mental health and resilience. Health issues, in particular psychological/psychiatric problems, were often a significant factor in dropout. Many students had been struggling with depression for many years, some taking leave of absence while they were unwell, and resuming their studies when able, only to ultimately end up leaving in the later part of the programme.

#### Absenteeism

Variables such as leave of absence, absenteeism, and homesickness have not been closely studied before. The high rate of documented absenteeism (30%) in our study is an important finding and may represent only the tip of the iceberg.

#### Social isolation

Our findings on social isolation are in keeping with other studies [8]. UK studies have shown that students living in campus accommodation had markedly lower dropout rates [12]. However, the majority of our overseas and First Year students live in University accommodation and this did not appear to protect against dropout.

### Factors associated with early and late dropout –year of programme

Academic difficulty (the main reason) and wrong career choice were the commonest reasons for dropout in the early years of the programme (Table 5). Our finding of higher dropout in the pre-clinical years, particularly First Year (60% dropouts in First Year), is in keeping with other studies [3]. This high attrition in First Year is not surprising. Students aged nineteen may lack the maturity to make informed career choices and may have difficulty adapting to the self-directed learning environment of University and to living away from home. Many students struggle with loneliness and homesickness. Others are unprepared for the volume of work. Some students quickly realise that they have made the wrong career choice and make an early decision to change courses. Only one student in our study cited parental influences to study medicine (although students may be slow to reveal this). Physical and psychological problems occur in students at all stages of the programme, but earlier on in the programme may not be revealed or may have been coped with by taking time-out.

Late dropout was associated with persistent academic failure, and psychological/physical ill-health, in particular, depression. Health problems tended to have been present for some time but may only have come to attention in later years. Students suffering from mental illness including addictions may have been reluctant to seek help for fear of ‘black-marking’.

The dropout rate in Final Year is low (5%) but dropout at this part of the programme is particularly traumatic and may reflect a failure to identify, support and offer alternative career strategies to students at an earlier stage in the course. Exiting the course in Final Year is particularly devastating and every effort must be made to identify and support students at risk of dropout before they progress to Final Year.

Our findings of 16% of dropouts occuring in Year 3 is higher than other studies [3]. Dropout in this year of the programme may be related to student commencement of full-time clinical rotations. The realities of a medical career may become apparent; students may be uncomfortable in clinical settings and may feel isolated from their colleagues.

#### English fluency problems

Good English fluency is important for both academic and social integration [36]. Although all students met the requisite English language requirements, English fluency problems were documented in seven (10%) dropout students (four of these were from Kuwait).

### Personal impact of dropout

Our study allowed us an insight into the personal impact of dropout, and events leading up to dropout, areas which have not received much attention. Of particular concern was the degree of social isolation (20% of dropout students). We identified recurrent themes of loneliness, failure, and despair, Dropout can be very traumatic for individual students who, having made the decision to study medicine, find that they cannot cope or realise that medicine was not the right choice.

### Attendance at student welfare services

Only one third of dropout students attended Student Welfare Services which is a cause for concern, as these are the very students who need help. Students may be afraid to disclose sensitive personal or health-related information because of worries about confidentiality. These students are also high achievers and may be reluctant to accept that they are having academic problems and that they need help.

### What courses did these students change to?

In contrast to other dropout studies where Science was the most common alternate career choice [4], Law was the most frequent career choice in our study.

### Dropout rate – comparison with other studies

Our attrition rate is relatively low (6.8% (53/779) and 5.7% (45/779) when students who transferred were excluded) compared to other studies [2,4,6-8,11,12,14-16,19,21,22,26,28,31,33,37-39]. A recent meta-analysis found an average attrition rate of 11.1% (2.4–26.2%) [3].

### Students who transferred to other medical programmes

In contrast to other dropout students, transfer students had a good academic record. Family and personal reasons were the usual reasons (students wanted to live near family). Most transferred after First Year, and some after the preclinical programme. Over half (6/11) of transfers were North American.

### Strengths and limitations

The strength of this study lies in the study’s detailed analysis of student factors associated with dropout (commonly studied variables along with other variables such as financial problems, social isolation, absenteeism, leave of absence) on a large cohort of students using a combination of qualitative and quantitative data.

The high incidence of absenteeism and leave of absence in our study suggests that these should be considered red flag signals and should be more closely monitored in student populations.

Importantly, this study identified a much higher rate of psychological illness (40%) in dropout students than previously described and deserves attention in all medical schools.

Our study showed a nationality-specific increased risk of dropout, suggesting the importance of analysing dropout rates for specific nationalities, rather than simply grouping students as ‘Overseas students’.

Another strength of the study was that it looked at the individual stories behind dropout allowing insight into the personal and emotional impact of dropout, an area where there has been little research. Qualitative analysis and inclusion of previously unstudied variables combined to gave a ‘big picture’ approach to determining the complex reasons underlying dropout. Behind every dropout statistic lies a personal story, some more tragic than others.

Another strength of the study was the analysis of the impact of changing the curriculum on attrition, a previously unstudied area.

The study also allowed us to compare dropout rates between a school-leaver programme and a Graduate-Entry programme, both programmes having similar teaching facilities/faculty. Results showed that dropout was lower in the Graduate-Entry programme, although this was not statistically significant. To date, only one cohort has completed the Graduate-Entry programme and student numbers are small.

Our study findings are of interest to all medical schools, as being able to identify students at risk of dropout is a concern for medical faculty in all institutions. Many medical schools are introducing new curricula and new Graduate-Entry programmes and are also increasing their intake of overseas students. Awareness of the effects of dropout on medical students is important for all medical schools.

#### What this study adds

•Our study looks at the personal stories behind dropout and impact of dropout on individual students.

•Students from some non-EU countries may be more at risk of dropout than other non-EU countries. suggesting a specific nationality-related risk of dropout.

•Social isolation is common in dropout students, and overseas students in particular may have problems with social integration and homesickness.

•Absenteeism is an important red flag signal in drop-out students and warrants early interview.

•There is a high prevalence of psychological problems amongst dropout students. Students suffering from physical, psychological and academic problems are slow to access Student Welfare services.

•Changing the curriculum does not appear to have an important effect on dropout.

•Graduate-Entry medical programmes, delivered in the same institution and by the same Faculty as school-leaver programmes, have a lower dropout rate than school-leaver programmes.

### Study limitations

Limitations of the study relate to restricted statistical analysis (lack of comparative data for the general medical student population).

This study was conducted in one medical school and our data may not necessarily generalise to other medical schools with different selection methods and student profiles. However, a similar list of underlying reasons for dropout has been found in international dropout studies, suggesting that our results may be relevant for other medical schools and can be generalised [5,20].

Causes of dropout may be multifactorial and reasons for withdrawal may be more complex than we were able to ascertain.

Recording of data was based on information in the student files which depended on what the student disclosed. It is very likely that the prevalence of some problems such as psychological/physical illness, family/personal problems etc. in the dropout population is higher than our findings. Especially in the case of psychiatric illness including addiction, eating disorders and depression, many cases may have been undiagnosed, unreported, or undocumented. We did, however, seek to supplement information by interviewing staff (this is subject to bias).

We were not able to analyse the effect of change in admission policy (more targeted selection processes are generally associated with lower dropout [22,32,33]) as none of the ‘HPAT’ cohorts have graduated yet.

We were unable to control for confounders when we analysed data for gender/nationality associations.

Qualitative analysis may have been prone to observer bias.

### Implications for future policy and research

Medical school attrition is important and there is a need for more rigorous studies and qualitative research on this subject [3].

Identification of institution factors (student selection, curriculum, course delivery and assessment) is an important research area and one which has not received much attention. Future studies need to address this. Future studies also need to examine the relationship between specific nationalities and rates of dropout.

Exit interviews should be considered in all medical schools.

Student Welfare services should be actively promoted, in particular to at-risk students (First Years, students who fail exams, and overseas students). Only a small proportion of our dropout students availed of Student Welfare services, despite the high prevalence of psychological ill-health and social isolation in this population. This suggests the need to regularly promote these supports to students and to reinforce the acceptability of accessing help. As numbers of non-EU students continue to expand, Student Welfare services are particularly important in this group. Students need to be reassured regarding confidentiality and the non-disclosure of health and social problems.

Mental Health services need to receive more attention in medical schools. Stress management programmes and programmes to increase awareness of depression and anxiety disorders should be promoted. Practical considerations regarding ongoing student health issues may need to be addressed by Fitness to Practice procedures.

It is important to identify language fluency problems at an early stage and arrange appropriate intervention.

Absenteeism should be viewed as an important red flag signal and prompt early interview. Consideration should be given to more vigilant attendance documentation.

Educational interventions (including key strategic learning skills)/communications skills/procedural skills) may be of benefit to some students with academic difficulty but research is necessary to evaluate the effects of these interventions [34]. Students who have difficulty with procedural skills or communication skills should be identified early in the programme.

Dropping out of medical school can be a very traumatic time in a young person’s life. It is important that we continue to support these students after they have left the programme and assist them in exploring other career options.

## Conclusions

Medical school attrition is important and needs to be monitored. Cause of dropout is often multifactorial and complex. Many students find the academic workload too challenging and after recurrent examination failure, are required to leave. Other students become ill, either physically or psychologically, and others realise that they have made a wrong career choice. In some cases, the cause remains unknown. Some students drop out unexpectedly while others have been under the radar for some time.

While there is no distinct ‘dropout profile’, certain red flag signals may alert us to the risk of dropout. These include academic underperformance, absenteeism, social isolation, overseas origin, depression, leave of absence, and English fluency problems.

More research is needed on the effects of institutional changes on dropout (type of curriculum, assessment, educational interventions, admission methods).

Our findings of nationality-related increased risk of dropout, and the high incidence of psychological problems, absenteeism, leave of absence and social isolation, are likely to be relevant to other medical schools and deserve attention.

Behind every dropout statistic is a vulnerable young adult who has left the medical programme. All medical schools have a duty of care to identify and support students at risk of dropout and to support students who leave in identifying new career options.

## Competing interests

The authors declare that they have no competing interests.

## Authors’ contributions

SO’F conceived the study. All authors were involved in study design. SO’F, BM, HH and CS collected and inputted data. SO’F, BM and AK did data analysis. BM and SO’F drafted the article and all authors revised it critically for important intellectual content. All authors read and approved the final version of the manuscript for publication.

## Authors’ information

BM is a Lecturer in Clinical Science and Practice at University College Cork and is a member of the Student Welfare Committee. S’OF is Head of Medical Education at University College Cork and has published a number of papers on medical school selection methods.

## Pre-publication history

The pre-publication history for this paper can be accessed here:

http://www.biomedcentral.com/1472-6920/13/13/prepub

## References

[B1] Higher Education Statistics Agency. Accessed 31st January 2013. http://www.hesa.ac.uk/index.php?option=com_content&task=view&id=2064&Itemid=141

[B2] HPAT Ireland www.hpat-ireland.acer.edu.au Accessed August 2012

[B3] O’Neill LD Wallstedt B Eika B Hartvigsen J Factors associated with dropout in medical education: a literature review Med Educ 2011 45 440 454 10.1111/j.1365-2923.2010.03898.x 21426375

[B4] Simpson KH Budd K Medical student attrition: a 10-year survey in one medical school Med Educ 1996 30 3 172 178 10.1111/j.1365-2923.1996.tb00739.x 8949550

[B5] Yates J When did they leave and why? A retrospective case study of attrition on the Nottingham undergraduate medical course BMC Med Educ 2012 12 43 10.1186/1472-6920-12-43 22716903 PMC3461480

[B6] Gough HG Hall WB An attempt to predict graduation from medical school J Med Educ 1975 50 10 940 950 1159763 10.1097/00001888-197510000-00003

[B7] Yates J James D Risk factors for poor performance on the undergraduate medical course: cohort study at Nottingham University Med Educ 2007 41 65 73 10.1111/j.1365-2929.2006.02648.x 17209894

[B8] Ward AM Kamien M Lopez DG Medical career choice and practice location: early factors predicting course completion, career choice and practice location Med Educ 2004 38 239 248 10.1046/j.1365-2923.2004.01762.x 14996332

[B9] Blackman IR Darmawan IN Graduate-entry medical student variables that predict academic and clinical achievement Int Ed J 2004 4 4 30 41

[B10] Haq I Higham J Morris R Dacrel J Effect of ethnicity and gender on performance in undergraduate medical examinations Med Educ 2005 39 1126 1128 10.1111/j.1365-2929.2005.02319.x 16262808

[B11] Arulampalam W Naylor R Smith J Factors affecting the probability of first year medical student dropout in the UK: a logistic analysis for the intake cohorts of 1980–92 Med Educ 2004 3 5 492 503 15107083 10.1046/j.1365-2929.2004.01815.x

[B12] Arulampalam W Naylor RA Smith JP Dropping out of medical school in the UK: explaining the changes over ten years Med Educ 2007 41 4 385 394 10.1111/j.1365-2929.2007.02710.x 17430284

[B13] Huff KL Fang D When are students most at risk of encountering academic difficulty? A study of the 1992 matriculants to U.S. medical schools Acad Med 1999 74 4 454 460 10.1097/00001888-199904000-00047 10219232

[B14] Neame RL Powis DA Bristow T Should medical students be selected only from recent school-leavers who have studied science? Med Educ 1992 26 433 440 10.1111/j.1365-2923.1992.tb00202.x 1461159

[B15] Fitzpatrick KM Wright MP Gender differences in medical school attrition rates, 1973–1992 J Am Med Womens Assoc 1995 50 6 204 206 8720396

[B16] Fogleman BY Zwagg RV Demographic, situational, and scholastic factors in medical school attrition South Med J 1981 74 5 602 606 10.1097/00007611-198105000-00025 7244720

[B17] Schmidt HG Cohen-Schotanus J Arends LR Impact of problem-based, active learning on graduation rates for 10 generations of Dutch medical students Med Educ 2009 43 211 218 10.1111/j.1365-2923.2008.03287.x 19250347

[B18] Hendren RL Predicting success and failure of medical students at risk for dismissal J Med Educ 1988 63 8 596 602 3398014 10.1097/00001888-198808000-00002

[B19] Lazin R Neumann L Student characteristics as predictors of drop-out from medical school: admissions to Beer-Sheva over a decade Med Educ 1991 25 396 404 10.1111/j.1365-2923.1991.tb00087.x 1758316

[B20] Powis DA Waring TC Bristow T O’Connell DL The structured interview as a tool for predicting premature withdrawal from medical school Aust N Z J Med 1992 22 6 692 698 10.1111/j.1445-5994.1992.tb04872.x 1489293

[B21] Strayhorn G Participation in a pre-medical summer programme for under-represented minority students as a predictor of academic performance in the first three years of medical school: two studies Acad Med 1999 74 4 435 447 10.1097/00001888-199904000-00043 10219228

[B22] Urlings-Strop LC Stijnen T Themmen APN Splinter TAW Selection of medical students: a controlled experiment Med Educ 2009 43 2 175 183 10.1111/j.1365-2923.2008.03267.x 19161489

[B23] Willoughby TL Arnold L Calkins V Personal characteristics and achievements of medical students from urban and non-urban areas J Med Educ 1981 56 9 717 726 7277433 10.1097/00001888-198109000-00003

[B24] McManus IC Woolf K Dacre J The educational background and qualifications of UK medical students from ethnic minorities Med Educ 2008 8 21 24 10.1186/1472-6920-8-21 PMC2359745 18416818

[B25] Lambe P Bristow D Predicting medical student performance from attributes at entry: a latent class analysis Med Educ 2011 45 308 316 10.1111/j.1365-2923.2010.03897.x 21299605

[B26] Hojat M Gonnella JS Erdmann JB Veloski JJ The fate of medical students with different levels of knowledge: are the basic medical sciences relevant to physician competence? Adv Health Sci Educ Theory Pract 1996 1 3 179 196 10.1023/A:1018383708357 24179018

[B27] Ferguson E James D O’ Hehir F Sanders A Pilot study of the roles of personality, references, and personal statements in relation to performance over the five years of a medical degree BMJ 2003 326 429 432 10.1136/bmj.326.7386.429 12595384 PMC163931

[B28] Stetto JE Gackstetter GD Cruess DF Hooper TI Variables associated with attrition from uniformed services university of the health sciences medical school Mil Med 2004 169 2 102 107 15040628 10.7205/milmed.169.2.102

[B29] Wass V Roberts C Hoogenboom R Jones R Van der Vleuten C Effect of ethnicity on performance in a final objective structured clinical examination: qualitative and quantitative study BMJ 2003 326 800 803 10.1136/bmj.326.7393.800 12689978 PMC153100

[B30] Woolf K Cave J Greenhalgh T Dacre J Ethnic stereotypes and the underachievement of UK medical students from ethnic minorities: qualitative study BMJ 2008 337 1220 1225 10.1136/bmj.a1220 PMC2517162 18710846

[B31] Iputo JE Kwizera E Problem-based learning improves the academic performance of medical students in South Africa Med Educ 2005 39 4 388 393 10.1111/j.1365-2929.2005.02106.x 15813761

[B32] O’Neill L Hartvigsen J Wallstedt B Korsholm L Eika B Medical school dropout - testing at admission versus selection by highest grades as predictors Med Educ 2011 45 1111 1120 10.1111/j.1365-2923.2011.04057.x 21988626

[B33] Reibnegger G Caluba HC Ithaler D Manhal S Neges HM Smolle J Progress of medical students after open admission or admission based on knowledge tests Med Educ 2010 44 2 205 214 10.1111/j.1365-2923.2009.03576.x 20059671

[B34] Winston KA van der Vleuten CP Scherpbier AJ An investigation into the design and effectiveness of a mandatory cognitive skills programme for at-risk medical students Med Teach 2010 32 3 236 243 10.3109/01421590903197035 20218839

[B35] Johnson DG The AAMC study of medical student attrition: overview and major findings J Med Educ 1965 40 10 913 920 5836640 10.1097/00001888-196510000-00001

[B36] Tinto V Clarke BR, Neave G Student attrition and retention Encyclopedia of higher education 1992 3 Oxford: Pergamon Press 1697 1709

[B37] Cohen-Schotanus J Muijtjens AM Reinders JJ Agsteribbe J van Rossum HJ van der Vleuten CP The predictive validity of grade point average scores in a partial lottery medical school admission system Med Educ 2006 40 10 1012 1019 10.1111/j.1365-2929.2006.02561.x 16987193

[B38] Parkhouse J Intake, output, and dropout in United Kingdom medical schools BMJ 1996 312 885 8611878 10.1136/bmj.312.7035.885 PMC2350578

[B39] Stegers-Jager KM Cohen-Schotanus J Splinter TA Themmen AP Academic dismissal policy for medical students:effect on study progress and help-seeking behaviour Med Educ 2011 45 987 994 10.1111/j.1365-2923.2011.04004.x 21883403

